# The American Academy of Medicine

**Published:** 1885-12

**Authors:** 


The American Academy of Medicine was organized in 1876, mainly with a view of encouraging a more liberal preliminary education of those contemplating the study of medicine, in the belief that this is the first and most important step toward securing a higher standard of medical education in the United States. The Academy has steadily grown until its membership now embraces about three hundred physicians, who have received the degree of Bachelor of Arts, in course, from recognized colleges or universities.

Having secured a recognized position, and exerted a beneficial influence on the medical profession of our country, it is believed by many members of the Academy that its sphere of usefulness may be still further extended by admitting to membership physicians who, have justly attained prominence in our profession, and, as an evidence thereof, and as a reward for good work accomplished, have received honorary degrees, from reputable institutions.

This enlargement was advocated by President Gihon in his annual address, at the last annual meeting of the Academy, in New York, in October, and the subject will be considered at the next annual meeting of the Academy, in Pittsburg, in September, 1886, just ten years after its organization.

